# The Secret (Mitotic) Life of Clathrin

**DOI:** 10.1111/tra.70047

**Published:** 2026-07-24

**Authors:** Stephen J. Royle

**Affiliations:** ^1^ Centre for Mechanochemical Cell Biology and Warwick Biomedical Sciences Warwick Medical School, University of Warwick Coventry UK

**Keywords:** Aurora‐a kinase, cancer, CKAP5, clathrin‐coated vesicles, endocytosis, GTSE1, mitosis, mitotic spindle, moonlighting, TACC3

## Abstract

The discovery of clathrin was a foundational event in membrane traffic research. Its identification as the major protein component of the coat that surrounds endocytic vesicles kickstarted the biochemical and molecular characterization of endocytosis. During this explosive period of cell biology, there was a parallel storyline involving clathrin which developed more slowly. It emerged that clathrin has an alternative function during mitosis, and that this function is unrelated to its membrane trafficking role. Clathrin forms part of a multiprotein complex that stabilizes microtubules of the mitotic spindle during the chromosome segregation events that occur during cell division. Due to this dual functionality, clathrin is sometimes referred to as a “moonlighting” protein. In this Perspective, we will take a look at this secret life of clathrin and examine how it carries out its mitotic function.

## Graphical abstract

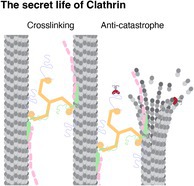

A perspective on the function of clathrin at the mitotic spindle during cell division.

## 1 Introduction

Fifty years ago, Barbara Pearse identified clathrin as the main component of the coat that surrounds endocytic vesicles, and kicked off the biochemical age of membrane traffic research [1]. Its name—clathrin—was given in reference to the lattice‐like structure of the vesicle coat which had been observed by electron microscopy since the mid‐1960s [2]. Following this, adaptors and other accessory proteins were identified and cloned as the field entered the molecular age. By the early 2000s, a whole suite of proteins involved in the process of coated vesicle formation had been discovered [3]. However, there was another side to clathrin: a second, mitotic function that emerged, more slowly but in parallel with the description of its function in membrane traffic.

In this Perspective, we'll examine this mitotic function of clathrin: covering what we know about its role in stabilizing microtubules and what questions remain about the secret life of clathrin.

## 2 Discovery of Clathrin's Mitotic Function

In the mid‐1980s, the first polyclonal antibodies raised against clathrin were isolated and used to localize the protein via indirect immunofluorescence microscopy. While these reagents revealed clathrin's localization in coated pits and vesicles at the light microscope level, Maro et al. reported that clathrin also decorated the second metaphase spindle in unfertilized mouse eggs [4]. This observation could have been due to non‐specific staining or a peculiarity of the cells studied; however, later work which used other antibodies to clathrin heavy chain or light chains in two different cell lines clearly showed that clathrin localizes to the microtubules (MTs) of the mitotic spindle during metaphase in somatic cell lines [5]. Besides this immunofluorescence microscopy evidence, clathrin was also identified as a mitotic spindle protein in the first attempts to proteomically characterize the mitotic spindle [6]. Later, using GFP‐tagged clathrin heavy chain or light chain, clathrin was again shown to be associated with the mitotic spindle [7]. Specifically, clathrin binds kinetochore microtubules (kMTs) that attach to the kinetochore on the mitotic chromosome and control chromosome movements during mitosis.

Given clathrin's membrane traffic role, the simplest explanation for the mitotic spindle localization was that kMTs have a large store of clathrin‐coated vesicles associated with them. However, mitotic spindles are largely free of membranes and there was no evidence for such a store at the electron microscopy level [8]. With seemingly no connection to membrane traffic, this raised the possibility of a novel function for clathrin at the mitotic spindle. Accordingly, when clathrin heavy chain was depleted using RNAi, mitotic defects were observed. The cells experienced a mitotic delay that was caused by destabilization of the kMTs, which resulted in prolonged spindle checkpoint signaling and a prolonged prometaphase [7].

Kinetochores are large multiprotein assemblies at the centromere of the chromosome which attach to kMTs. Each kinetochore has many kMTs attached, and these are bundled together as a fiber. Between each MT are a series of crosslinking proteins that were proposed to bundle the kMTs and give stability to the fiber [9]. The N‐terminal domain—or foot—of clathrin was required for kMT binding, because deletion mutants failed to localize to the spindle [7]. Since clathrin is a three‐legged protein, and therefore has more than one MT binding region, it was a candidate for conferring stability to the fiber by crosslinking MTs [7]. Indeed, interfering with trimerization affected the mitotic function of clathrin which agreed with this model [10]. Together the localization, proteomic and functional data pointed to a mitotic function for clathrin that was independent of its role in membrane traffic. Even at this early stage, it seemed unlikely that clathrin acted alone at the mitotic spindle, and so the next step was to identify which protein(s) clathrin associated with at the mitotic spindle and to determine how this protein complex binds to MTs. Work in the years that followed led to the identification of the molecular players and on to our current model of the molecular details of the mitotic clathrin complex.

## 3 Molecular Details of the Mitotic Clathrin Complex

**FIGURE 1:**
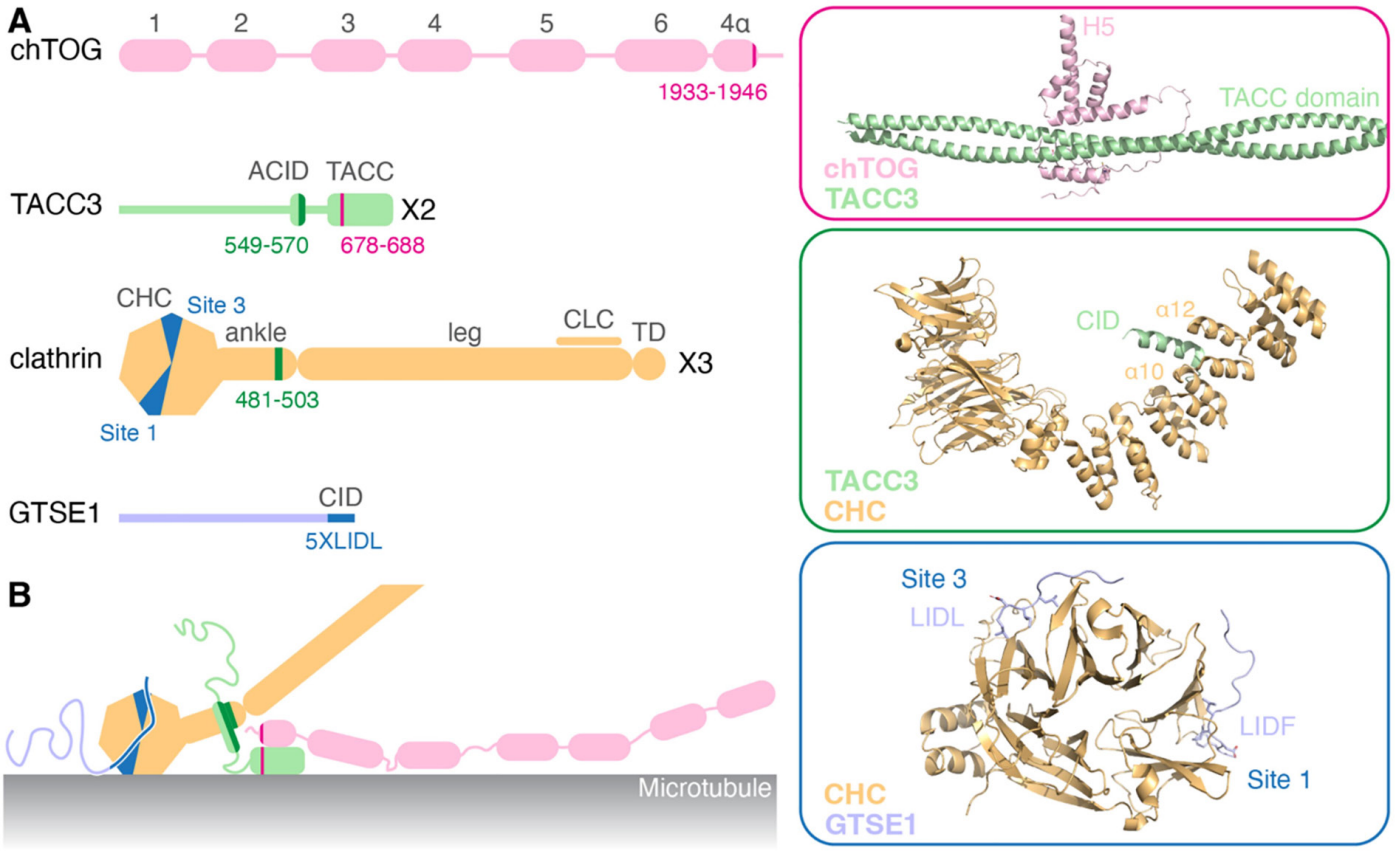
Structural details of the mitotic clathrin complex. (A) Primary domain layout of chTOG, TACC3, clathrin GTSE1. Interaction regions between chTOG–TACC3, TACC3–CHC and GTSE1–CHC are indicated in dark pink, green and blue, respectively. ACID, Aurora‐A–clathrin interaction domain; CHC, clathrin heavy chain; CID, clathrin interaction domain; CLC, clathrin light chain. Right: structural details of the interactions within the complex. PDB codes: 5ODS (TACC3/clathrin), 6QNP (GTSE1/clathrin), chTOG/TACC3 interaction was generated by AlphaFold3. (B) Schematic diagram of how the complex likely binds to a microtubule. The interaction surface is composed of the CHC N‐terminal domain and the TACC domain of TACC3. GTSE1 and chTOG are ancilliary to the microtubule interaction.

The current picture of the spindle clathrin complex is that there are four components: (i) clathrin, (ii) TACC3, (iii) GTSE1, and (iv) chTOG/CKAP5. These components were identified by several groups, using a variety of different approaches and systems [11, 12, 13, 14]. We now know that the complex is formed by three pairwise interactions: (i) TACC3 binds clathrin, (ii) chTOG binds TACC3, and (iii) GTSE1 binds clathrin (Figure 1), and that its formation is governed by mitotic phosphorylation. When all four proteins are together as a complex, they can bind to the lattice of MTs in kinetochore fibers.

### 3.1 TACC3–Clathrin

During mitosis, phosphorylation of TACC3 on serine 558 by Aurora‐A kinase controls the interaction between TACC3 and clathrin [11, 15, 16, 17, 18]. This interaction is unusual because clathrin does not have a phospho‐reader domain that would typically receive a phosphorylated residue. Instead, the phosphorylation of S558 on TACC3 promotes a helical conformation of an otherwise disordered region [18]. Helices 8 to 10 of clathrin heavy chain (CHC) repeat 0 (CHCR0) interact with a DPLL motif in TACC3, while the CHC residue K507 selects the phosphorylated form of TACC3 to bind but is not involved directly in the interaction [19]. The binding of TACC3 to clathrin is thought to coordinate the N‐terminal domain of CHC so that it is adjacent to the TACC domain of TACC3 in order to make a composite microtubule‐binding surface for the complex. In support of this idea, this composite arrangement could be mimicked by a direct fusion of these two domains, which could then bind MTs in a phospho‐independent manner [17].

Aurora‐A kinase activity is crucial to complex formation. Inhibition of the kinase causes the complex to disassemble and fall off microtubules [17]. It also explains why this complex is specific to mitosis and is not assembled during interphase since Aurora‐A kinase is under cell cycle control.

The current model is that clathrin and TACC3 are the core of the complex, anchoring it to the MTs with GTSE1 and chTOG acting as ancillary subunits whose presence does not affect microtubule binding [20]. This model is surprising given that both GTSE1 and chTOG have some affinity for microtubules [21, 22]. However, without their respective interactions with clathrin and TACC3, neither GTSE1 nor chTOG are localized to spindle microtubules [20]. While the model of joint TACC3–clathrin binding to MTs is the current view, there are reports that some residual clathrin binding to spindle MTs is seen when TACC3 is depleted by RNAi in somatic cells [12, 14] and oocytes [23] or when TACC3 is replaced in chicken DT40 cells with a mutant that cannot bind clathrin [24].

### 3.2 chTOG–TACC3

The interaction between chTOG and TACC3 occurs via a “stutter” in the dimeric coiled‐coil (TACC) domain of TACC3 and a helical domain in the C‐terminal region of chTOG [17, 25]. An α‐helix (H5) that is normally stacked against a four‐helix bundle when chTOG is alone moves away and becomes part of a parallel trimeric coiled‐coil in the stutter region of the TACC domain [26]. The helical bundle region of chTOG is distinct from the rest of the molecule, which comprises five TOG domains and a sixth TOG‐like domain (Figure 1). These TOG domains can bind tubulin dimers and are important for the MT polymerization activity of chTOG [27].

### 3.3 GTSE1–Clathrin

The third pairwise interaction in the complex is between GTSE1 and clathrin. It occurs via linear peptide motifs in the GTSE1 intrinsically disordered C‐terminal domain and the seven‐bladed beta propeller N‐terminal domain of CHC [28]. GTSE1 has five motifs that are similar to clathrin box motifs (CBMs) in this region and it is the fourth (LIDL) that binds to site 3 on CHC, while site 1 is occupied by either the second, third, or fifth CBMs (Figure 1). The clathrin NTD sites 1 and 3 are better known to membrane traffickers as the binding sites for the first identified CBM (LΦXΦ[DE]) and arrestin ([LI][LI]GXL) [29]. The fact that these membrane traffic sites are repurposed for clathrin's mitotic function nicely illustrates how the two functions of clathrin—in membrane traffic and in mitosis—are indeed distinct [28].

## 4 Mitotic Function of the Clathrin Complex

**FIGURE 2:**
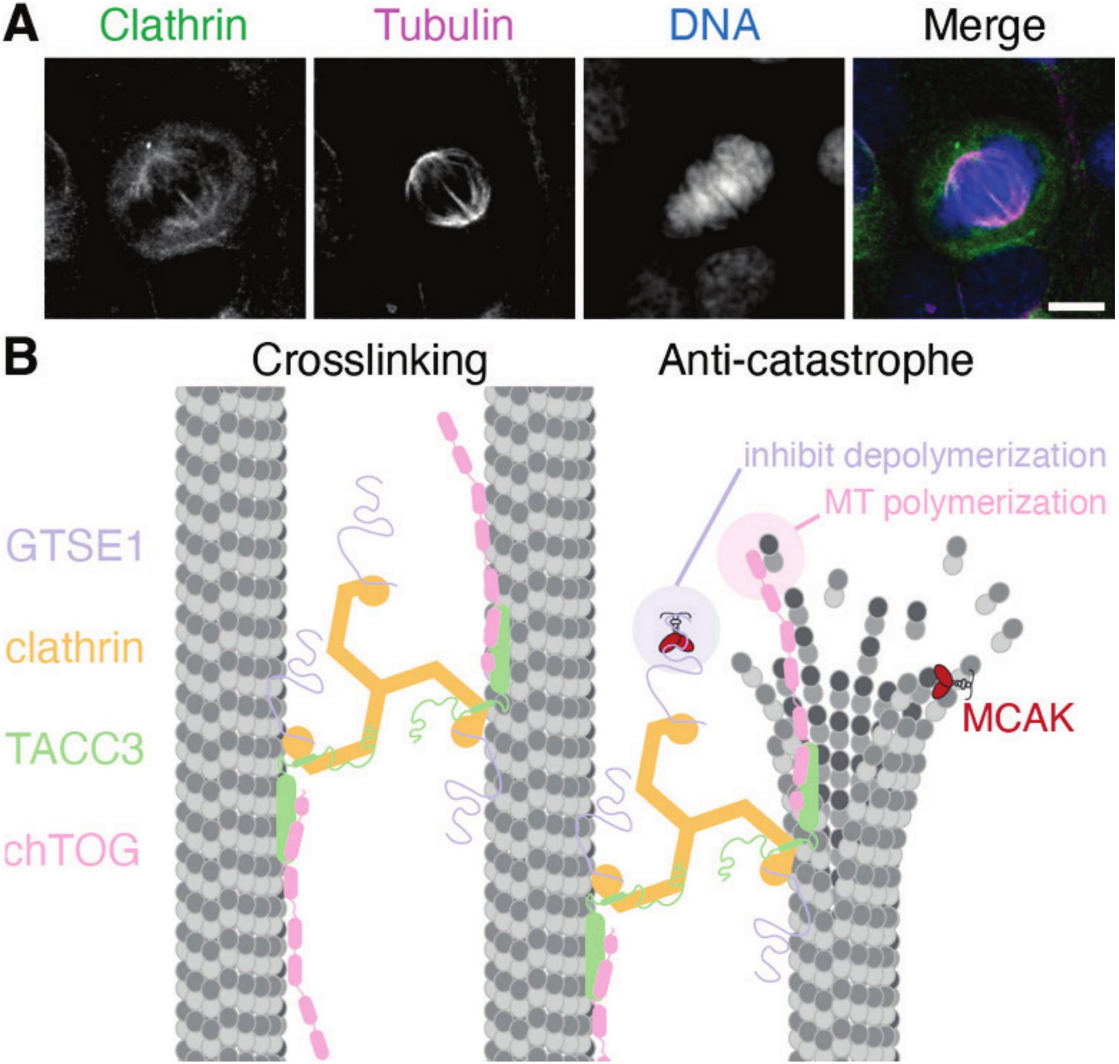
The mitotic function of clathrin. (A) Clathrin at the mitotic spindle in a HeLa cell. Maximum intensity projection of a formaldehyde‐fixed HeLa cell in mitosis stained for clathrin heavy chain (green), tubulin (magenta) and DNA (blue), scale bar, 10 μm. (B) Kinetochore fiber stabilization. 1. Kinetochore MTs are crosslinked by the mitotic clathrin complex. 2. The complex has anti‐catastrophe properties by (a) GTSE1 inhibiting the activity of the MT depolymerase MCAK and (b) promoting MT growth via the MT polymerase chTOG.

As described above, the initial idea was that the complex strengthens and stabilizes the K‐fibers, because clathrin is a three‐legged protein, and therefore the complex can crosslink microtubules within the fiber. However, it seems likely that the complex also acts as an anti‐catastrophe factor (Figure 2). MTs undergo cycles of polymerization and depolymerization, sometimes undergoing catastrophe where the MT completely shrinks. Proteins that can prevent this depolymerization are known as anti‐catastrophe factors.

The crosslinking function, put forward in 2005 [7], is underpinned by the following observations: First, trimerization of clathrin is required for proper mitotic function [10]. Second, clathrin could be identified as electron densities between adjacent kMTs [11] and later it was visualized in 3D tomograms as a web of contacts that was termed “the mesh” [30]. Third, loss of the complex from kMTs results in mitotic defects including destabilized K‐fibers [7, 11, 13, 14, 15, 20]. Fourth, the purified clathrin complex could bundle microtubules in vitro [30].

If the function of the mitotic clathrin complex is solely to act as a MT crosslinker, and GTSE1 and chTOG are not required for MT binding, then the question is what are they doing in the complex? Mutation of site 1 on the N‐terminal domain of CHC impairs mitosis [17], although additional mutations render clathrin unable to bind the spindle, it points to an active role of GTSE1 in the clathrin complex because it binds to clathrin via this site. Indeed [28] showed that clathrin's recruitment of GTSE1 to kMTs contributes to their stability by antagonizing the activity of KIF2C/MCAK, a major microtubule depolymerase [31]. TACC3 had previously been demonstrated to stabilize MTs by antagonizing the depolymerase activity of MCAK and this was proposed to be via the MT polymerase chTOG [32]. Whether these activities are shared between chTOG and GTSE1 or via GTSE1 alone, it seems clear that the mitotic clathrin complex promotes mitotic microtubule growth (or counteracts MT depolymerization) and that the overall stabilization effect is a consequence of MT crosslinking and MT polymerization.

The difficulty in nailing down the mitotic function of the clathrin complex is due to two things. First, mitosis is a complex orchestration of many different proteins, where upsetting any player has consequences on multiple players and potentially all subsequent steps of cell division. Second, the four components of the clathrin complex each participate in other complexes and activities in a variety of subcellular localizations [25] (Table 1).

**TABLE 1:** Subcellular localization(s) of members of the spindle clathrin complex.

Protein or complex	Interphase	Mitosis
Clathrin	Clathrin‐coated pits [3]	Clathrin‐coated pits [33]
chTOG	Centrosomes [34]	Centrosomes [34], Kinetochores [22]
GTSE1	MT plus‐ends (with EB1) [21], Nucleus [35]	Astral MTs [36]
TACC3	Nucleus [37]	None
TACC3‐chTOG	MT plus‐ends [38]	MT plus‐ends [25], PCM [26]
Clathrin‐TACC3‐chTOG‐GTSE1	None	kMTs [20]

During mitosis, chTOG binds to kinetochores, independently of microtubules, via an interaction with Hec1, where it plays a role in mitotic error correction [22]. TACC3 and chTOG, together, track the growing ends of microtubules in the spindle and in interphase cells [25, 38, 39]. Separately, GTSE1 also tracks the plus ends of microtubules by binding EB1 [21, 36]. This activity is restricted to interphase and is inhibited during mitosis due to CDK1 phosphorylation. At the spindle pole, chTOG localizes to the centrosome while TACC3 is observed close by [25]. Of course, clathrin is found in coated pits and vesicles; although during mitosis endocytosis is suppressed, coated pits are still present [33, 40, 41]. These localizations and a few others not mentioned here are summarized in Table 1.

Knocking out or knocking down a component of the mitotic clathrin complex is likely to affect a number of other functions during mitosis, and also in interphase. One approach has been to rapidly remove components using induced relocalization or “knocksideways” [15, 20], where a tagged protein is relocalized to a neutral location (the mitochondria) and thereby inactivated [42]. While this circumvents the chronic effects of depletion [43], all complexes or subcomplexes which contain the tagged protein are removed. Therefore, recent work has centered on finding specific inhibitors for each of the interactions in the complex.

Several different inhibitors of clathrin's NTD binding sites have been developed in the hope of finding an inhibitor of clathrin‐mediated endocytosis [44]. Such compounds would be predicted to interfere with GTSE1 binding during mitosis and potentially destabilize mitotic spindle microtubules, a strategy that could be used as an anti‐cancer treatment. While the first described chemical inhibitors of the clathrin NTD had issues with off‐target effects and cytotoxicity, the latest generation of compounds looks promising [45]. The first‐generation compounds were reported to impact mitosis [46], although the latest agents have yet to be tested for any effect in cell division. A related strategy is to use a peptide (Wbox2) as a competitive inhibitor to displace proteins that bind the NTD [29]. Again, this approach is sufficient to inhibit endocytosis, but has yet to be tested in mitosis.

The TACC3–chTOG interaction has been targeted using an Affimer, a small, engineered binding protein. Affimers that selectively bind the stutter in the TACC3 TACC domain were able to compete with chTOG for binding and therefore act as competitive inhibitors of this specific interaction [26]. These tools helped to uncover a novel function for the TACC3–chTOG subcomplex in spindle pole integrity, which was independent of the mitotic clathrin complex.

Finally, inhibitors targeting the clathrin–TACC3 interaction have recently been developed. These stapled peptide reagents mimic the structure of the helix on TACC3‐pS558 which usually interacts with CHC [19]. Moreover, the peptides are cell permeable and therefore can enter cells and disrupt the clathrin–TACC3 interaction, which results in a delay in mitotic progression. Further optimization of these reagents is therefore a promising route to interfere with cell division. One disadvantage of using MT drugs in cancer treatment is that MTs are important for normal cell function in addition to their mitotic function, so side effects are a significant issue. Targeting the spindle clathrin complex—which is only assembled during mitosis—is potentially a cleaner route to interfere with mitosis in cancer cells.

## 5 Other Membrane Traffic Proteins with Functions in Mitosis

The realization that clathrin has a function in mitosis—a time when membrane traffic is suppressed—led to speculation that other membrane traffic proteins might be similarly repurposed. The lipid kinase PI3K‐C2α/PIK3C2A was implicated as a fifth member of the mitotic clathrin complex [12, 47], due to its co‐purification with TACC3, clathrin and GTSE1 [12]. It was proposed that PIK3C2A strengthens the clathrin‐TACC3 interaction [47], by binding both partners in a non‐enzymatic, structural role. However, there are three reasons to doubt this model. First, other membrane traffic proteins (CLINT1, SEC13, SEC16A) also co‐purified with TACC3, clathrin and GTSE1 [12] with cross‐purification of vesicles possibly explaining this result [48]. Second, the interaction site for PIK3C2A on clathrin overlaps with the GTSE1 interaction site, which raises the question of whether both proteins can be present in the complex [28]. Third, there are conflicting reports of whether PIK3C2A is consistently observed at the mitotic spindle [20, 49]. In light of these reasons, it is doubtful therefore that PIK3C2A is an essential part of the complex, but it is possible that it may participate in a regulated way that is not yet understood.

There have been reports of many other membrane traffic proteins that have distinct roles during mitosis. Mitotic roles for dynamin‐2/DNM2 [50], Rab6A'/RAB6C [51, 52], epsin/EPN1 [53], ARH/LDLRAP1 [54], GAK [55, 56], ITSN2 [57], RALBP1 [58], and TRAPPC12 [59] have been proposed in centrosome cohesion, spindle assembly/orientation, checkpoint signaling, and kinetochore assembly. Although in many cases, these mitotic functions have not been characterized further beyond a single report, so it is unclear how essential or conserved these alternative roles may be.

Conversely, there are proteins that were first characterized with a mitotic role which also have a membrane traffic function. First, the mitotic checkpoint protein ZW10 is part of the RZZ complex (ROD–Zwilch–ZW10) that must be removed from kinetochores along with Mad2 by dynein in order for mitosis to proceed [60]. During interphase, ZW10 is part of the NRZ complex (NAG–RINT‐1–ZW10) and is involved in ER‐Golgi transport and the maintenance of Golgi morphology [61]; most likely through an interaction with cytoplasmic dynein [62]. Second, BUB1 and BUBR1 are core spindle assembly checkpoint proteins that appear to have discrete interphase functions in membrane traffic. BUBR1 binds the clathrin adaptor protein AP2 β2 subunit and modulates insulin receptor endocytosis in an interplay between Mad2, p31comet and insulin receptor substrates 1 and 2 [63, 64]. BUB1 meanwhile is implicated in EGFR internalization and down‐regulation [65], and in viral entry via endocytosis [66]. Third, a further kinetochore protein CENP‐F, has been implicated in mitochondrial trafficking in addition to chromosome segregation [67].

There is an important distinction between (i) proteins that are demonstrated to have two completely independent functions (moonlighting), and (ii) proteins with a single function where the perturbation of that function impacts mitosis. For example, RNAi screens of endocytic proteins reveal mitotic defects that are likely due to inhibition of membrane trafficking rather than distinct mitotic functions [68]. Similarly, caveolins influence mitotic spindle orientation, but this is due to caveolae being anchor points for astral microtubules, rather than a separate mitotic function for caveolins [69, 70]. There are also many membrane trafficking proteins that are involved in membrane remodeling during cytokinesis; these include ESCRT‐III components, EHD family proteins, Rabs, and SNAREs [71]. Perturbation studies where membrane dynamics, organelle remodeling, or cell rounding are altered will inevitably affect mitosis and/or cell division, and so care is required when interpreting such experiments.

## 6 Conclusion and Outlook

The notion that the entire repertoire of trafficking proteins is repurposed wholesale in mitosis has turned out not to be the case. Instead it seems that there are a small number of proteins that can be genuinely considered moonlighting proteins. Some have argued that these examples point to a deep connection between membrane traffic and mitosis, but it is unclear whether this connection between two essential eukaryotic functions is meaningful, or simply that moonlighting is a common evolutionary strategy and these examples have been discovered due to intense research activity in these areas. After all, nuclear transport proteins are also reported to moonlight on the mitotic spindle [72], which indicates that the partnership between membrane traffic and mitosis might not be exclusive. There are several unresolved questions regarding clathrin's secret life in mitosis. First, how does the mitotic clathrin complex selectively bind kMTs versus other MTs in the mitotic spindle? Perhaps the complex detects parallel microtubules or it has a preference for the MT spacing within the kinetochore fiber. Second, what is the contribution of MT cross‐linking versus MT polymerization in the role of the clathrin complex in stabilizing kMTs? Third, when did this mitotic function for clathrin arise? Clathrin, TACC3 and chTOG are highly conserved proteins whereas GTSE1 is vertebrate‐specific; whether the function of the complex is conserved beyond vertebrates is currently unclear. Fourth, is molecular interference with clathrin complex formation a viable strategy to inhibit mitosis in a cancer context? There are likely cancer types that are more reliant on the action of this complex and their identification, as well as improved inhibition strategies, have promise for future treatment.

## Funding

This work was supported by Biotechnology and Biological Sciences Research Council (BB/V003062/1) and Human Frontier Science Program (HFSP RGP25/2022).

## Conflicts of Interest

The author declares no conflicts of interest.

## Data Availability

Data sharing not applicable to this article as no datasets were generated or analysed during the current study.
